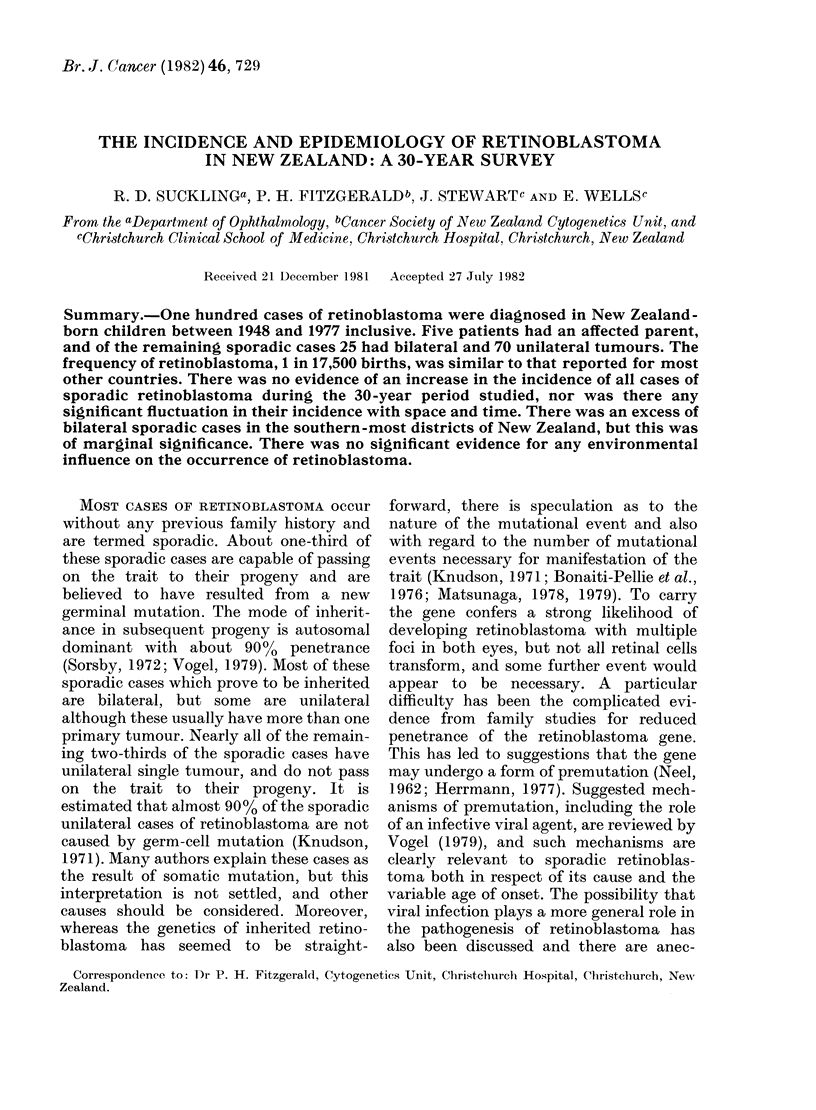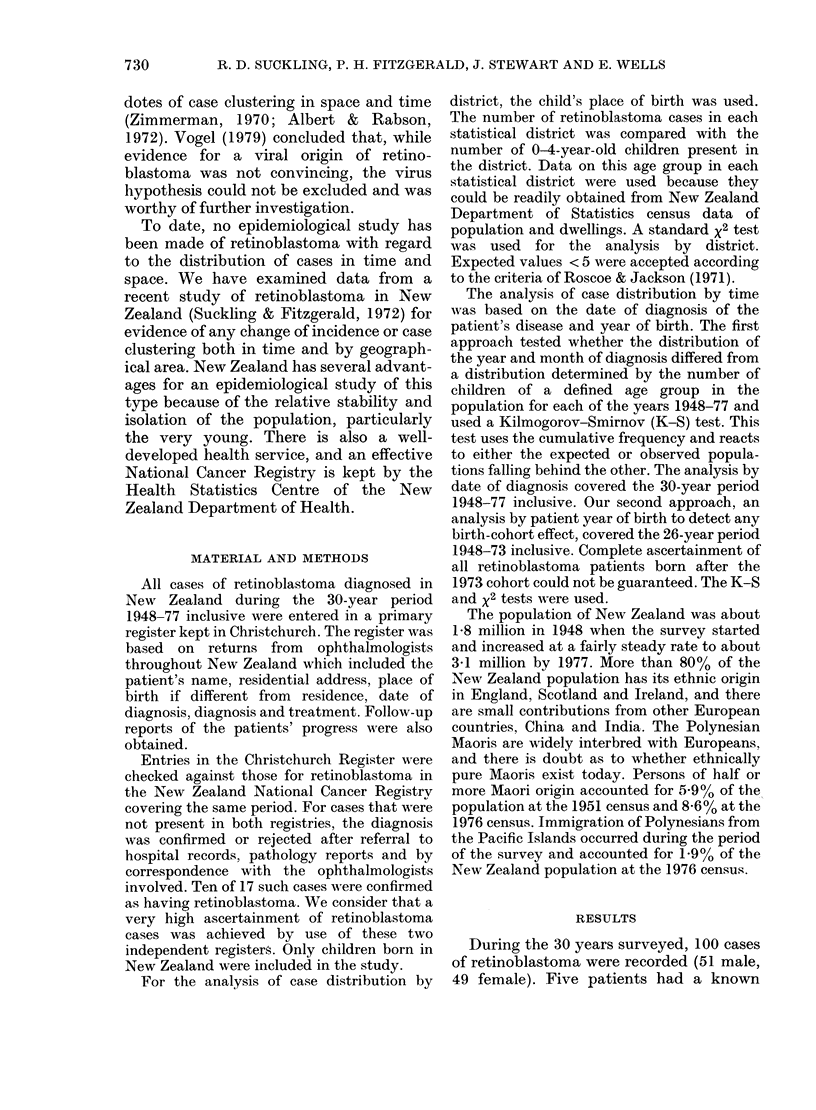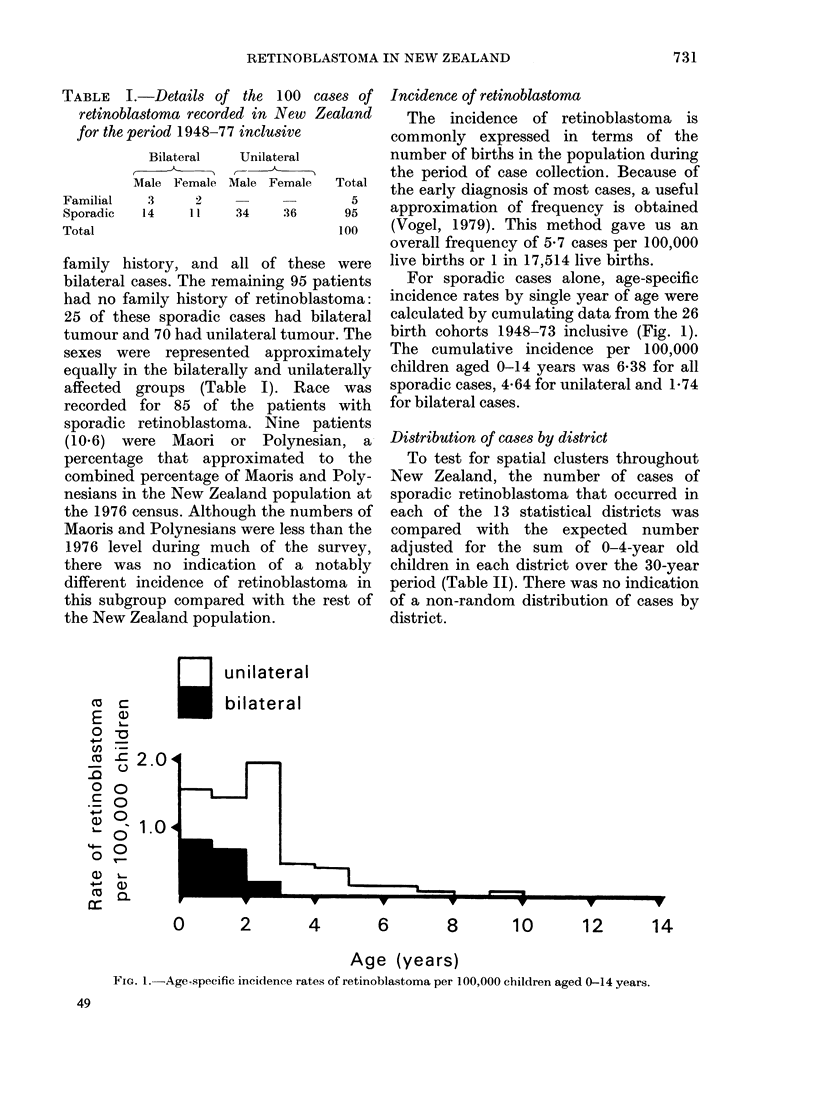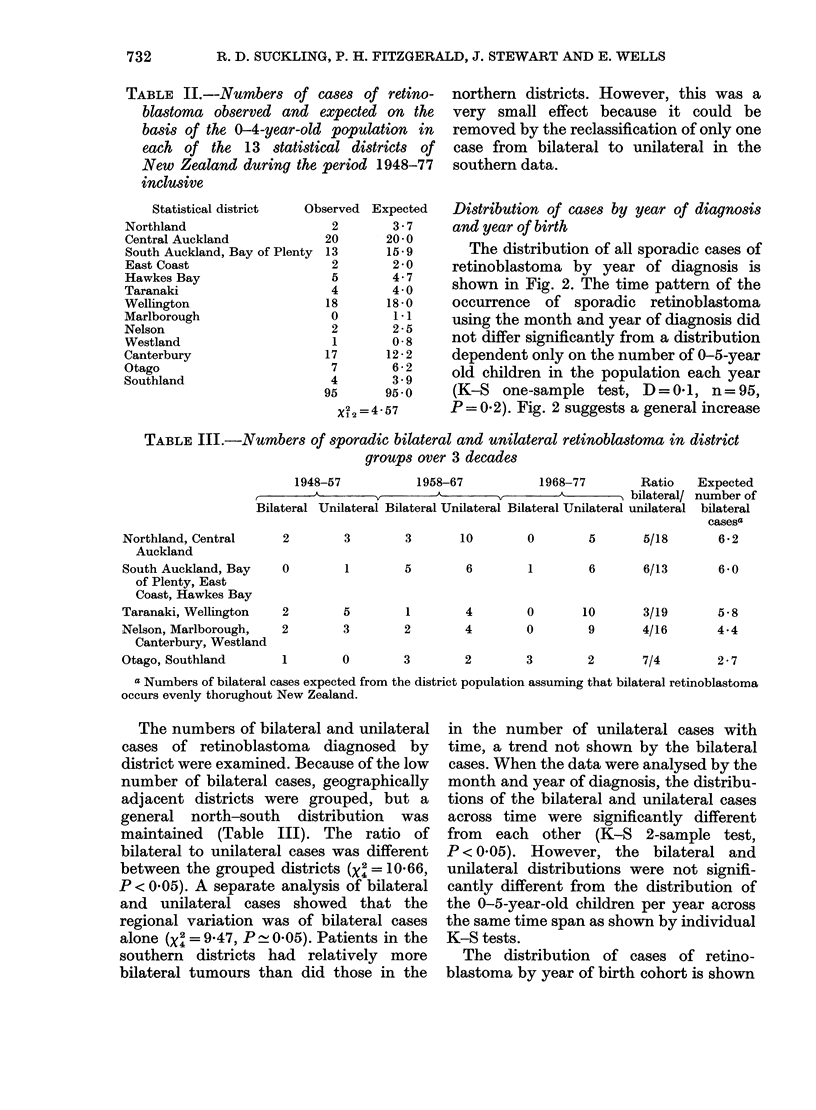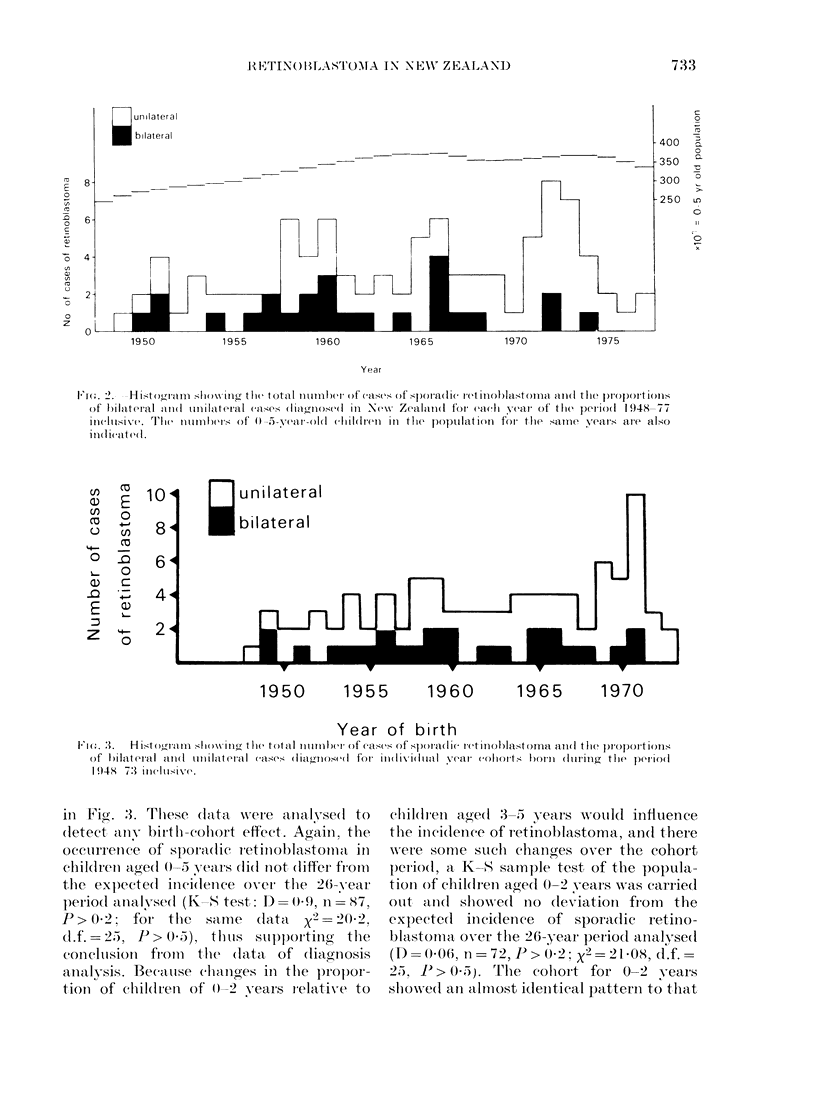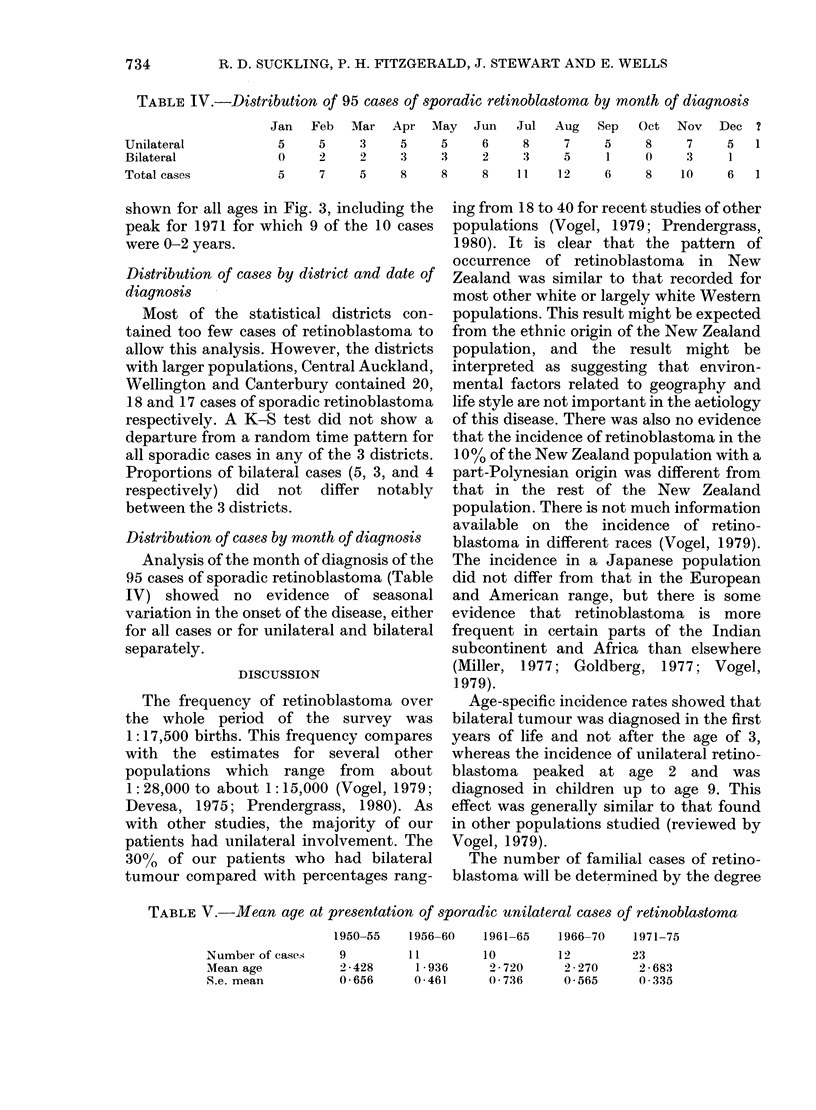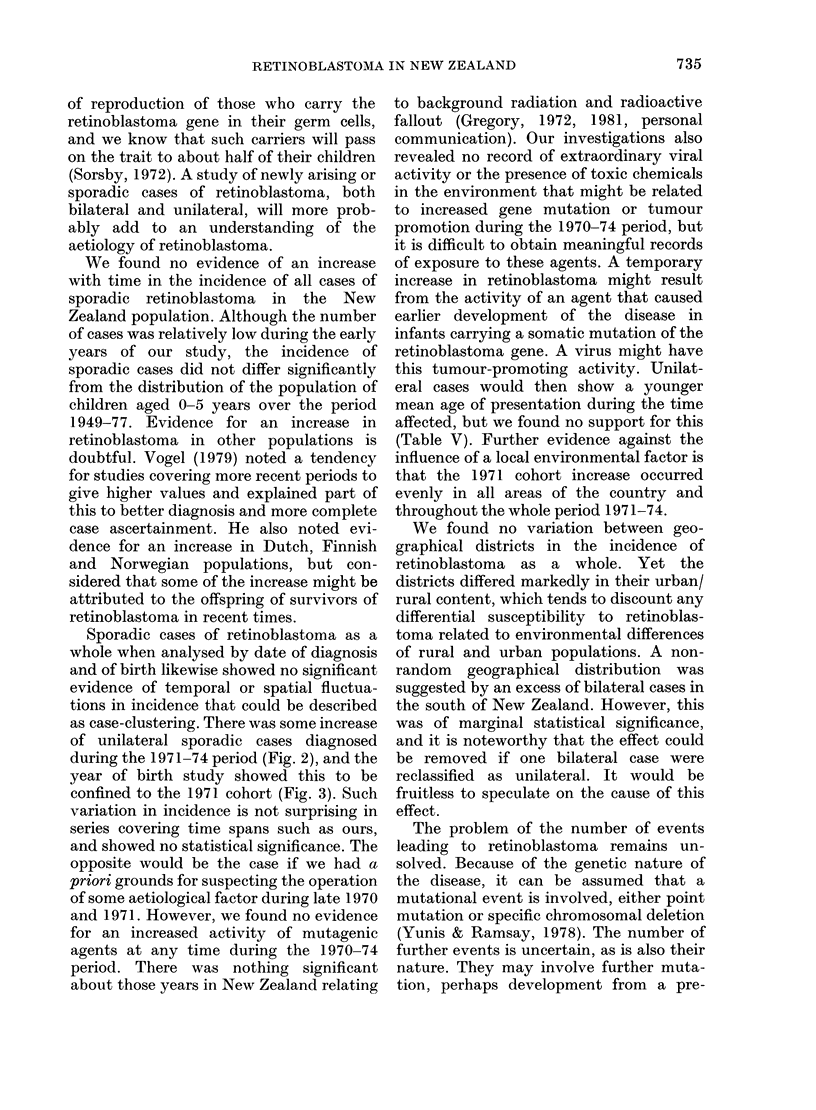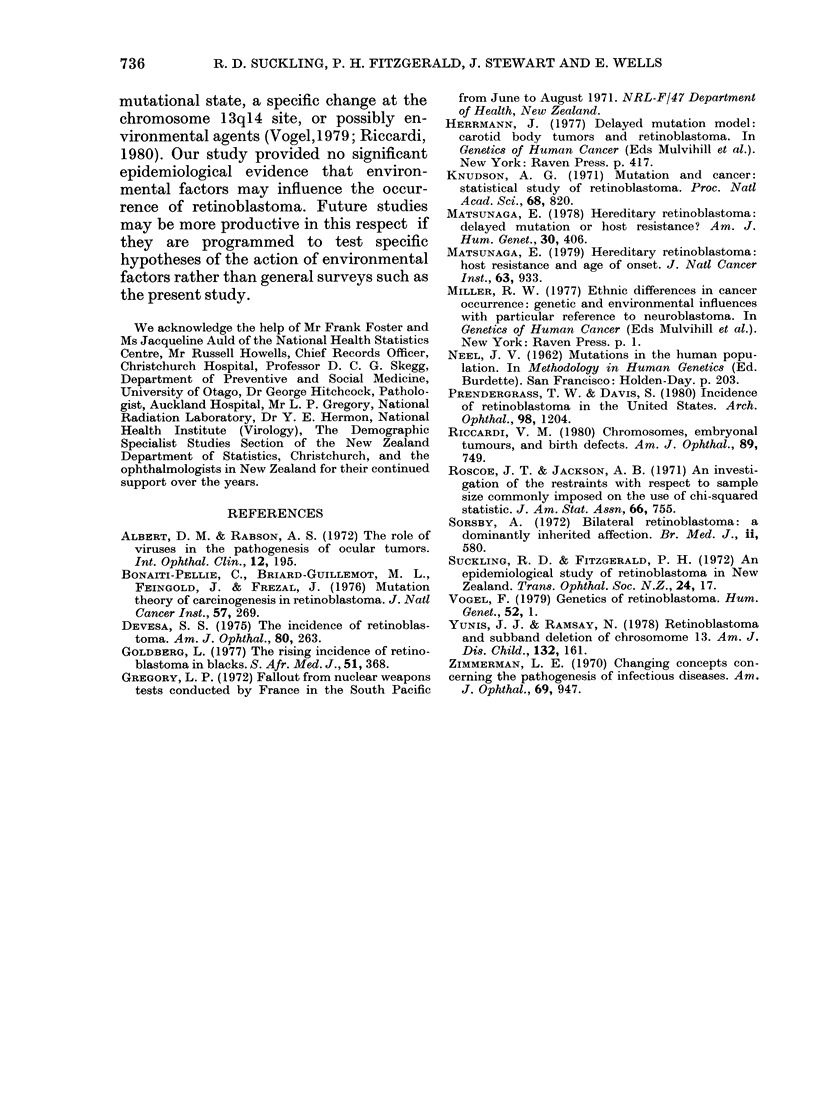# The incidence and epidemiology of retinoblastoma in New Zealand: A 30-year survey.

**DOI:** 10.1038/bjc.1982.265

**Published:** 1982-11

**Authors:** R. D. Suckling, P. H. Fitzgerald, J. Stewart, E. Wells

## Abstract

One hundred cases of retinoblastoma were diagnosed in New Zealand-born children between 1948 and 1977 inclusive. Five patients had an affected parent, and of the remaining sporadic cases 25 had bilateral and 70 unilateral tumours. The frequency of retinoblastoma, 1 in 17,500 births, was similar to that reported for most other countries. There was no evidence of an increase in the incidence of all cases of sporadic retinoblastoma during the 30-year period studied, nor was there any significant fluctuation in their incidence with space and time. There was an excess of bilateral sporadic cases in the southern-most districts of New Zealand, but this was of marginal significance. There was no significance evidence for any environmental influence on the occurrence of retinoblastoma.


Br. J. Cancer (1982) 46, 729

### THE INCIDENCE AND EPIDEMIOLOGY OF RETINOBLASTOMA IN NEW ZEALAND: A 30-YEAR SURVEY

R. D. SUCKLING[a], P. H. FITZGERALD[b], J. STEWART[c] AND E. WELLS[c]

*From the [a]Department of Ophthalmology, [b]Cancer Society of New Zealand Cytogenetics Unit, and [c]Christchurch Clinical School of Medicine, Christchurch Hospital, Christchurch, New Zealand*

Received 21 December 1981 Accepted 27 July 1982

**Summary.—One hundred cases of retinoblastoma were diagnosed in New Zealand-born children between 1948 and 1977 inclusive. Five patients had an affected parent, and of the remaining sporadic cases 25 had bilateral and 70 unilateral tumours. The frequency of retinoblastoma, 1 in 17,500 births, was similar to that reported for most other countries. There was no evidence of an increase in the incidence of all cases of sporadic retinoblastoma during the 30-year period studied, nor was there any significant fluctuation in their incidence with space and time. There was an excess of bilateral sporadic cases in the southern-most districts of New Zealand, but this was of marginal significance. There was no significant evidence for any environmental influence on the occurrence of retinoblastoma.**

MOST CASES OF RETINOBLASTOMA occur without any previous family history and are termed sporadic. About one-third of these sporadic cases are capable of passing on the trait to their progeny and are believed to have resulted from a new germinal mutation. The mode of inheritance in subsequent progeny is autosomal dominant with about 90% penetrance (Sorsby, 1972; Vogel, 1979). Most of these sporadic cases which prove to be inherited are bilateral, but some are unilateral although these usually have more than one primary tumour. Nearly all of the remaining two-thirds of the sporadic cases have unilateral single tumour, and do not pass on the trait to their progeny. It is estimated that almost 90% of the sporadic unilateral cases of retinoblastoma are not caused by germ-cell mutation (Knudson, 1971). Many authors explain these cases as the result of somatic mutation, but this interpretation is not settled, and other causes should be considered. Moreover, whereas the genetics of inherited retinoblastoma has seemed to be straightforward, there is speculation as to the nature of the mutational event and also with regard to the number of mutational events necessary for manifestation of the trait (Knudson, 1971; Bonaiti-Pellie *et al.*, 1976; Matsunaga, 1978, 1979). To carry the gene confers a strong likelihood of developing retinoblastoma with multiple foci in both eyes, but not all retinal cells transform, and some further event would appear to be necessary. A particular difficulty has been the complicated evidence from family studies for reduced penetrance of the retinoblastoma gene. This has led to suggestions that the gene may undergo a form of premutation (Neel, 1962; Herrmann, 1977). Suggested mechanisms of premutation, including the role of an infective viral agent, are reviewed by Vogel (1979), and such mechanisms are clearly relevant to sporadic retinoblastoma both in respect of its cause and the variable age of onset. The possibility that viral infection plays a more general role in the pathogenesis of retinoblastoma has also been discussed and there are anec-

Correspondence to: Dr P. H. Fitzgerald, Cytogenetics Unit, Christchurch Hospital, Christchurch, New Zealand.

dotes of case clustering in space and time (Zimmerman, 1970; Albert & Rabson, 1972). Vogel (1979) concluded that, while evidence for a viral origin of retinoblastoma was not convincing, the virus hypothesis could not be excluded and was worthy of further investigation.

To date, no epidemiological study has been made of retinoblastoma with regard to the distribution of cases in time and space. We have examined data from a recent study of retinoblastoma in New Zealand (Suckling & Fitzgerald, 1972) for evidence of any change of incidence or case clustering both in time and by geographical area. New Zealand has several advantages for an epidemiological study of this type because of the relative stability and isolation of the population, particularly the very young. There is also a well-developed health service, and an effective National Cancer Registry is kept by the Health Statistics Centre of the New Zealand Department of Health.

### MATERIAL AND METHODS

All cases of retinoblastoma diagnosed in New Zealand during the 30-year period 1948–77 inclusive were entered in a primary register kept in Christchurch. The register was based on returns from ophthalmologists throughout New Zealand which included the patient's name, residential address, place of birth if different from residence, date of diagnosis, diagnosis and treatment. Follow-up reports of the patients' progress were also obtained.

Entries in the Christchurch Register were checked against those for retinoblastoma in the New Zealand National Cancer Registry covering the same period. For cases that were not present in both registries, the diagnosis was confirmed or rejected after referral to hospital records, pathology reports and by correspondence with the ophthalmologists involved. Ten of 17 such cases were confirmed as having retinoblastoma. We consider that a very high ascertainment of retinoblastoma cases was achieved by use of these two independent registers. Only children born in New Zealand were included in the study.

For the analysis of case distribution by district, the child's place of birth was used. The number of retinoblastoma cases in each statistical district was compared with the number of 0–4-year-old children present in the district. Data on this age group in each statistical district were used because they could be readily obtained from New Zealand Department of Statistics census data of population and dwellings. A standard $\chi^2$ test was used for the analysis by district. Expected values $< 5$ were accepted according to the criteria of Roscoe & Jackson (1971).

The analysis of case distribution by time was based on the date of diagnosis of the patient's disease and year of birth. The first approach tested whether the distribution of the year and month of diagnosis differed from a distribution determined by the number of children of a defined age group in the population for each of the years 1948–77 and used a Kilmogorov–Smirnov (K–S) test. This test uses the cumulative frequency and reacts to either the expected or observed populations falling behind the other. The analysis by date of diagnosis covered the 30-year period 1948–77 inclusive. Our second approach, an analysis by patient year of birth to detect any birth-cohort effect, covered the 26-year period 1948–73 inclusive. Complete ascertainment of all retinoblastoma patients born after the 1973 cohort could not be guaranteed. The K–S and $\chi^2$ tests were used.

The population of New Zealand was about 1·8 million in 1948 when the survey started and increased at a fairly steady rate to about 3·1 million by 1977. More than 80% of the New Zealand population has its ethnic origin in England, Scotland and Ireland, and there are small contributions from other European countries, China and India. The Polynesian Maoris are widely interbred with Europeans, and there is doubt as to whether ethnically pure Maoris exist today. Persons of half or more Maori origin accounted for 5·9% of the population at the 1951 census and 8·6% at the 1976 census. Immigration of Polynesians from the Pacific Islands occurred during the period of the survey and accounted for 1·9% of the New Zealand population at the 1976 census.

### RESULTS

During the 30 years surveyed, 100 cases of retinoblastoma were recorded (51 male, 49 female). Five patients had a known

TABLE I.—*Details of the* 100 *cases of retinoblastoma recorded in New Zealand for the period* 1948–77 *inclusive*

| | Bilateral | | Unilateral | | |
|---|---|---|---|---|---|
| | Male | Female | Male | Female | Total |
| Familial | 3 | 2 | — | — | 5 |
| Sporadic | 14 | 11 | 34 | 36 | 95 |
| Total | | | | | 100 |

family history, and all of these were bilateral cases. The remaining 95 patients had no family history of retinoblastoma: 25 of these sporadic cases had bilateral tumour and 70 had unilateral tumour. The sexes were represented approximately equally in the bilaterally and unilaterally affected groups (Table I). Race was recorded for 85 of the patients with sporadic retinoblastoma. Nine patients (10·6) were Maori or Polynesian, a percentage that approximated to the combined percentage of Maoris and Polynesians in the New Zealand population at the 1976 census. Although the numbers of Maoris and Polynesians were less than the 1976 level during much of the survey, there was no indication of a notably different incidence of retinoblastoma in this subgroup compared with the rest of the New Zealand population.

*Incidence of retinoblastoma*

The incidence of retinoblastoma is commonly expressed in terms of the number of births in the population during the period of case collection. Because of the early diagnosis of most cases, a useful approximation of frequency is obtained (Vogel, 1979). This method gave us an overall frequency of 5·7 cases per 100,000 live births or 1 in 17,514 live births.

For sporadic cases alone, age-specific incidence rates by single year of age were calculated by cumulating data from the 26 birth cohorts 1948–73 inclusive (Fig. 1). The cumulative incidence per 100,000 children aged 0–14 years was 6·38 for all sporadic cases, 4·64 for unilateral and 1·74 for bilateral cases.

*Distribution of cases by district*

To test for spatial clusters throughout New Zealand, the number of cases of sporadic retinoblastoma that occurred in each of the 13 statistical districts was compared with the expected number adjusted for the sum of 0–4-year old children in each district over the 30-year period (Table II). There was no indication of a non-random distribution of cases by district.

FIG. 1.—Age-specific incidence rates of retinoblastoma per 100,000 children aged 0–14 years.

TABLE II.—*Numbers of cases of retinoblastoma observed and expected on the basis of the 0–4-year-old population in each of the* 13 *statistical districts of New Zealand during the period* 1948–77 *inclusive*

| Statistical district | Observed | Expected |
|---|---|---|
| Northland | 2 | 3·7 |
| Central Auckland | 20 | 20·0 |
| South Auckland, Bay of Plenty | 13 | 15·9 |
| East Coast | 2 | 2·0 |
| Hawkes Bay | 5 | 4·7 |
| Taranaki | 4 | 4·0 |
| Wellington | 18 | 18·0 |
| Marlborough | 0 | 1·1 |
| Nelson | 2 | 2·5 |
| Westland | 1 | 0·8 |
| Canterbury | 17 | 12·2 |
| Otago | 7 | 6·2 |
| Southland | 4 | 3·9 |
| | 95 | 95·0 |
| | $\chi^2_{12} = 4 \cdot 57$ | |

northern districts. However, this was a very small effect because it could be removed by the reclassification of only one case from bilateral to unilateral in the southern data.

*Distribution of cases by year of diagnosis and year of birth*

The distribution of all sporadic cases of retinoblastoma by year of diagnosis is shown in Fig. 2. The time pattern of the occurrence of sporadic retinoblastoma using the month and year of diagnosis did not differ significantly from a distribution dependent only on the number of 0–5-year old children in the population each year (K–S one-sample test, $D = 0{\cdot}1$, $n = 95$, $P = 0{\cdot}2$). Fig. 2 suggests a general increase in the number of unilateral cases with time, a trend not shown by the bilateral cases. When the data were analysed by the month and year of diagnosis, the distributions of the bilateral and unilateral cases across time were significantly different from each other (K–S 2-sample test, $P < 0{\cdot}05$). However, the bilateral and unilateral distributions were not significantly different from the distribution of the 0–5-year-old children per year across the same time span as shown by individual K–S tests.

The distribution of cases of retinoblastoma by year of birth cohort is shown

TABLE III.—*Numbers of sporadic bilateral and unilateral retinoblastoma in district groups over* 3 *decades*

| | 1948–57 | | 1958–67 | | 1968–77 | | Ratio bilateral/ unilateral | Expected number of bilateral cases[a] |
|---|---|---|---|---|---|---|---|---|
| | Bilateral | Unilateral | Bilateral | Unilateral | Bilateral | Unilateral | | |
| Northland, Central Auckland | 2 | 3 | 3 | 10 | 0 | 5 | 5/18 | 6·2 |
| South Auckland, Bay of Plenty, East Coast, Hawkes Bay | 0 | 1 | 5 | 6 | 1 | 6 | 6/13 | 6·0 |
| Taranaki, Wellington | 2 | 5 | 1 | 4 | 0 | 10 | 3/19 | 5·8 |
| Nelson, Marlborough, Canterbury, Westland | 2 | 3 | 2 | 4 | 0 | 9 | 4/16 | 4·4 |
| Otago, Southland | 1 | 0 | 3 | 2 | 3 | 2 | 7/4 | 2·7 |

[a] Numbers of bilateral cases expected from the district population assuming that bilateral retinoblastoma occurs evenly thorughout New Zealand.

The numbers of bilateral and unilateral cases of retinoblastoma diagnosed by district were examined. Because of the low number of bilateral cases, geographically adjacent districts were grouped, but a general north–south distribution was maintained (Table III). The ratio of bilateral to unilateral cases was different between the grouped districts ($\chi^2_4 = 10{\cdot}66$, $P < 0{\cdot}05$). A separate analysis of bilateral and unilateral cases showed that the regional variation was of bilateral cases alone ($\chi^2_4 = 9{\cdot}47$, $P \simeq 0{\cdot}05$). Patients in the southern districts had relatively more bilateral tumours than did those in the in Fig. 3. These data were analysed to detect any birth-cohort effect. Again, the occurrence of sporadic retinoblastoma in children aged 0–5 years did not differ from the expected incidence over the 26-year period analysed (K–S test: D = 0·9, n = 87, $P > 0{\cdot}2$; for the same data $\chi^2 = 20{\cdot}2$, d.f. = 25, $P > 0{\cdot}5$), thus supporting the conclusion from the data of diagnosis analysis. Because changes in the proportion of children of 0–2 years relative to children aged 3–5 years would influence the incidence of retinoblastoma, and there were some such changes over the cohort period, a K–S sample test of the population of children aged 0–2 years was carried out and showed no deviation from the expected incidence of sporadic retinoblastoma over the 26-year period analysed (D = 0·06, n = 72, $P > 0{\cdot}2$; $\chi^2 = 21{\cdot}08$, d.f. = 25, $P > 0{\cdot}5$). The cohort for 0–2 years showed an almost identical pattern to that

TABLE IV.—*Distribution of 95 cases of sporadic retinoblastoma by month of diagnosis*

| | Jan | Feb | Mar | Apr | May | Jun | Jul | Aug | Sep | Oct | Nov | Dec | ? |
|---|---|---|---|---|---|---|---|---|---|---|---|---|---|
| Unilateral | 5 | 5 | 3 | 5 | 5 | 6 | 8 | 7 | 5 | 8 | 7 | 5 | 1 |
| Bilateral | 0 | 2 | 2 | 3 | 3 | 2 | 3 | 5 | 1 | 0 | 3 | 1 | |
| Total cases | 5 | 7 | 5 | 8 | 8 | 8 | 11 | 12 | 6 | 8 | 10 | 6 | 1 |

shown for all ages in Fig. 3, including the peak for 1971 for which 9 of the 10 cases were 0–2 years.

*Distribution of cases by district and date of diagnosis*

Most of the statistical districts contained too few cases of retinoblastoma to allow this analysis. However, the districts with larger populations, Central Auckland, Wellington and Canterbury contained 20, 18 and 17 cases of sporadic retinoblastoma respectively. A K–S test did not show a departure from a random time pattern for all sporadic cases in any of the 3 districts. Proportions of bilateral cases (5, 3, and 4 respectively) did not differ notably between the 3 districts.

*Distribution of cases by month of diagnosis*

Analysis of the month of diagnosis of the 95 cases of sporadic retinoblastoma (Table IV) showed no evidence of seasonal variation in the onset of the disease, either for all cases or for unilateral and bilateral separately.

### DISCUSSION

The frequency of retinoblastoma over the whole period of the survey was 1:17,500 births. This frequency compares with the estimates for several other populations which range from about 1:28,000 to about 1:15,000 (Vogel, 1979; Devesa, 1975; Prendergrass, 1980). As with other studies, the majority of our patients had unilateral involvement. The 30% of our patients who had bilateral tumour compared with percentages ranging from 18 to 40 for recent studies of other populations (Vogel, 1979; Prendergrass, 1980). It is clear that the pattern of occurrence of retinoblastoma in New Zealand was similar to that recorded for most other white or largely white Western populations. This result might be expected from the ethnic origin of the New Zealand population, and the result might be interpreted as suggesting that environmental factors related to geography and life style are not important in the aetiology of this disease. There was also no evidence that the incidence of retinoblastoma in the 10% of the New Zealand population with a part-Polynesian origin was different from that in the rest of the New Zealand population. There is not much information available on the incidence of retinoblastoma in different races (Vogel, 1979). The incidence in a Japanese population did not differ from that in the European and American range, but there is some evidence that retinoblastoma is more frequent in certain parts of the Indian subcontinent and Africa than elsewhere (Miller, 1977; Goldberg, 1977; Vogel, 1979).

Age-specific incidence rates showed that bilateral tumour was diagnosed in the first years of life and not after the age of 3, whereas the incidence of unilateral retinoblastoma peaked at age 2 and was diagnosed in children up to age 9. This effect was generally similar to that found in other populations studied (reviewed by Vogel, 1979).

The number of familial cases of retinoblastoma will be determined by the degree

TABLE V.—*Mean age at presentation of sporadic unilateral cases of retinoblastoma*

| | 1950–55 | 1956–60 | 1961–65 | 1966–70 | 1971–75 |
|---|---|---|---|---|---|
| Number of cases | 9 | 11 | 10 | 12 | 23 |
| Mean age | 2·428 | 1·936 | 2·720 | 2·270 | 2·683 |
| S.e. mean | 0·656 | 0·461 | 0·736 | 0·565 | 0·335 |

of reproduction of those who carry the retinoblastoma gene in their germ cells, and we know that such carriers will pass on the trait to about half of their children (Sorsby, 1972). A study of newly arising or sporadic cases of retinoblastoma, both bilateral and unilateral, will more probably add to an understanding of the aetiology of retinoblastoma.

We found no evidence of an increase with time in the incidence of all cases of sporadic retinoblastoma in the New Zealand population. Although the number of cases was relatively low during the early years of our study, the incidence of sporadic cases did not differ significantly from the distribution of the population of children aged 0–5 years over the period 1949–77. Evidence for an increase in retinoblastoma in other populations is doubtful. Vogel (1979) noted a tendency for studies covering more recent periods to give higher values and explained part of this to better diagnosis and more complete case ascertainment. He also noted evidence for an increase in Dutch, Finnish and Norwegian populations, but considered that some of the increase might be attributed to the offspring of survivors of retinoblastoma in recent times.

Sporadic cases of retinoblastoma as a whole when analysed by date of diagnosis and of birth likewise showed no significant evidence of temporal or spatial fluctuations in incidence that could be described as case-clustering. There was some increase of unilateral sporadic cases diagnosed during the 1971–74 period (Fig. 2), and the year of birth study showed this to be confined to the 1971 cohort (Fig. 3). Such variation in incidence is not surprising in series covering time spans such as ours, and showed no statistical significance. The opposite would be the case if we had *a priori* grounds for suspecting the operation of some aetiological factor during late 1970 and 1971. However, we found no evidence for an increased activity of mutagenic agents at any time during the 1970–74 period. There was nothing significant about those years in New Zealand relating to background radiation and radioactive fallout (Gregory, 1972, 1981, personal communication). Our investigations also revealed no record of extraordinary viral activity or the presence of toxic chemicals in the environment that might be related to increased gene mutation or tumour promotion during the 1970–74 period, but it is difficult to obtain meaningful records of exposure to these agents. A temporary increase in retinoblastoma might result from the activity of an agent that caused earlier development of the disease in infants carrying a somatic mutation of the retinoblastoma gene. A virus might have this tumour-promoting activity. Unilateral cases would then show a younger mean age of presentation during the time affected, but we found no support for this (Table V). Further evidence against the influence of a local environmental factor is that the 1971 cohort increase occurred evenly in all areas of the country and throughout the whole period 1971–74.

We found no variation between geographical districts in the incidence of retinoblastoma as a whole. Yet the districts differed markedly in their urban/rural content, which tends to discount any differential susceptibility to retinoblastoma related to environmental differences of rural and urban populations. A nonrandom geographical distribution was suggested by an excess of bilateral cases in the south of New Zealand. However, this was of marginal statistical significance, and it is noteworthy that the effect could be removed if one bilateral case were reclassified as unilateral. It would be fruitless to speculate on the cause of this effect.

The problem of the number of events leading to retinoblastoma remains unsolved. Because of the genetic nature of the disease, it can be assumed that a mutational event is involved, either point mutation or specific chromosomal deletion (Yunis & Ramsay, 1978). The number of further events is uncertain, as is also their nature. They may involve further mutation, perhaps development from a premutational state, a specific change at the chromosome 13q14 site, or possibly environmental agents (Vogel, 1979; Riccardi, 1980). Our study provided no significant epidemiological evidence that environmental factors may influence the occurrence of retinoblastoma. Future studies may be more productive in this respect if they are programmed to test specific hypotheses of the action of environmental factors rather than general surveys such as the present study.

We acknowledge the help of Mr Frank Foster and Ms Jacqueline Auld of the National Health Statistics Centre, Mr Russell Howells, Chief Records Officer, Christchurch Hospital, Professor D. C. G. Skegg, Department of Preventive and Social Medicine, University of Otago, Dr George Hitchcock, Pathologist, Auckland Hospital, Mr L. P. Gregory, National Radiation Laboratory, Dr Y. E. Hermon, National Health Institute (Virology), The Demographic Specialist Studies Section of the New Zealand Department of Statistics, Christchurch, and the ophthalmologists in New Zealand for their continued support over the years.

### REFERENCES

ALBERT, D. M. & RABSON, A. S. (1972) The role of viruses in the pathogenesis of ocular tumors. *Int. Ophthal. Clin.*, **12**, 195.

BONAITI-PELLIE, C., BRIARD-GUILLEMOT, M. L., FEINGOLD, J. & FREZAL, J. (1976) Mutation theory of carcinogenesis in retinoblastoma. *J. Natl Cancer Inst.*, **57**, 269.

DEVESA, S. S. (1975) The incidence of retinoblastoma. *Am. J. Ophthal.*, **80**, 263.

GOLDBERG, L. (1977) The rising incidence of retinoblastoma in blacks. *S. Afr. Med. J.*, **51**, 368.

GREGORY, L. P. (1972) Fallout from nuclear weapons tests conducted by France in the South Pacific from June to August 1971. *NRL-F/47 Department of Health, New Zealand.*

HERRMANN, J. (1977) Delayed mutation model: carotid body tumors and retinoblastoma. In *Genetics of Human Cancer* (Eds Mulvihill *et al.*). New York: Raven Press. p. 417.

KNUDSON, A. G. (1971) Mutation and cancer: statistical study of retinoblastoma. *Proc. Natl Acad. Sci.*, **68**, 820.

MATSUNAGA, E. (1978) Hereditary retinoblastoma: delayed mutation or host resistance? *Am. J. Hum. Genet.*, **30**, 406.

MATSUNAGA, E. (1979) Hereditary retinoblastoma: host resistance and age of onset. *J. Natl Cancer Inst.*, **63**, 933.

MILLER, R. W. (1977) Ethnic differences in cancer occurrence: genetic and environmental influences with particular reference to neuroblastoma. In *Genetics of Human Cancer* (Eds Mulvihill *et al.*). New York: Raven Press. p. 1.

NEEL, J. V. (1962) Mutations in the human population. In *Methodology in Human Genetics* (Ed. Burdette). San Francisco: Holden-Day. p. 203.

PRENDERGRASS, T. W. & DAVIS, S. (1980) Incidence of retinoblastoma in the United States. *Arch. Ophthal.*, **98**, 1204.

RICCARDI, V. M. (1980) Chromosomes, embryonal tumours, and birth defects. *Am. J. Ophthal.*, **89**, 749.

ROSCOE, J. T. & JACKSON, A. B. (1971) An investigation of the restraints with respect to sample size commonly imposed on the use of chi-squared statistic. *J. Am. Stat. Assn*, **66**, 755.

SORSBY, A. (1972) Bilateral retinoblastoma: a dominantly inherited affection. *Br. Med. J.*, **ii**, 580.

SUCKLING, R. D. & FITZGERALD, P. H. (1972) An epidemiological study of retinoblastoma in New Zealand. *Trans. Ophthal. Soc. N.Z.*, **24**, 17.

VOGEL, F. (1979) Genetics of retinoblastoma. *Hum. Genet.*, **52**, 1.

YUNIS, J. J. & RAMSAY, N. (1978) Retinoblastoma and subband deletion of chrosomome 13. *Am. J. Dis. Child.*, **132**, 161.

ZIMMERMAN, L. E. (1970) Changing concepts concerning the pathogenesis of infectious diseases. *Am. J. Ophthal.*, **69**, 947.